# Restoring redox homeostasis through S-adenosyl-L-methionine and B-vitamins co-supplementation alleviates ethanol induced hepato-pancreatic injury by regulating glutathione biosynthesis in C57BL/6J mice

**DOI:** 10.3389/fphar.2026.1761748

**Published:** 2026-04-22

**Authors:** Prasanth Babu Nandagopal, Venkatraman Manickam

**Affiliations:** School of Biosciences and Technology, Vellore Institute of Technology (VIT), Vellore, Tamil Nadu, India

**Keywords:** antioxidant system, chronic ethanol exposure, co-supplementation, Lieber-DeCarli liquid diet, oxidative stress, S-adenosyl-L-methionine

## Abstract

**Background:**

Alcohol use disorder(AUD) is a chronic condition responsible for more than 3 million deaths annually worldwide and contributes to the development of multiple systemic comorbidities. Chronic alcohol consumption promotes inflammatory responses, immune suppression, antioxidant dysregulation, and nutritional deficiencies, severely affecting organs such as the liver and pancreas. Disruption of methionine metabolism and antioxidant defenses, including reduced availability of S-adenosyl-L-methionine (SAMe) and essential B-vitamins, has been implicated in alcohol-induced oxidative stress. Therefore, the aim of the present study was to investigate whether co-supplementation with SAMe and B-vitamins could alleviate ethanol-induced hepato-pancreatic injury by improving redox homeostasis and glutathione biosynthesis.

**Methods:**

C57BL/6J male mice (n = 6 per group) were used to model acute-on-chronic alcohol-associated liver injury using the NIAAA chronic-plus-binge ethanol feeding model. From days 1–10, mice were fed a Lieber-DeCarli liquid diet containing 5% (v/v) ethanol *(ad libitum)* to establish chronic ethanol exposure while receiving co-supplementation with SAMe(5 mg/kg) and B-vitamins (B6-7 mg/kg; B12-50 μg/kg; B1-3.68 mg/kg). On day 11, mice received a single ethanol dose of 5 g/kg body weight by oral gavage as an acute binge ethanol exposure. Liver and pancreatic function was assessed by quantifying serum enzymatic markers. Oxidative and redox status was evaluated through biochemical estimation of nitric oxide, lipid peroxidation, hydrogen sulfide, and glutathione levels. Inflammatory responses were assessed by TNF-α quantification and NF-κB (p65) DNA-binding activity, while histopathological alterations were examined using H&E staining. Additionally, the expression of key genes (*Nos2, Gclc, Gpx1, and Cyp2e1*) were analysed to corroborate the findings.

**Results:**

SAMe and B-vitamin co-supplementation significantly attenuated ethanol-induced hepato-pancreatic injury, evidenced by reduced activities of amylase, alanine aminotransferase, and aspartate aminotransferase, myeloperoxidase (*p* < 0.001) and promoted restoration of tissue architecture. Inflammatory signalling was markedly suppressed, reflected by decreased NF-κB and TNF-α levels (*p* < 0.05), alongside a significant reduction in oxidative stress markers, including nitric oxide, lipid peroxidation, and *Cyp2e1* expression (*p* < 0.001), collectively indicating a protective therapeutic effect.

**Conclusion:**

Chronic ethanol exposure induces profound oxidative stress and impairs endogenous antioxidant defence system, culminating in severe hepato-pancreatic injury. Restoration of glutathione homeostasis and stabilization of cellular redox balance through SAMe and B-vitamins co-supplementation efficiently attenuated organ pathology, thereby improving the overall wellbeing of the affected individual.

## 1 Introduction

Alcohol consumption remains a pervasive global health challenge, contributing significantly to morbidity and mortality worldwide (Park and Kim, 2020). While moderate consumption may carry nuanced health implications, excessive and chronic alcohol intake exerts detrimental effects on nearly every organ system, majorly driving the body towards a persistent state of low-grade inflammation with elevated levels of oxidative stress (Tan et al., 2020; Ambade and Mandrekar, 2012). This toxic ambiance arises primarily from the metabolism of ethanol itself, which generates reactive oxygen species (ROS) and toxic byproducts like acetaldehyde, disrupting cellular homeostasis and triggering pro-inflammatory cascades (González-Reimers et al., 2014). The liver, as the primary site of alcohol metabolism, endures the initial and most significant toxic burden of chronic alcohol consumption (Osna et al., 2017). The resulting pathology is a progressive disease continuum, beginning with hepatic steatosis (fatty liver) and advancing to steatohepatitis, fibrosis, cirrhosis, and potentially hepatocellular carcinoma. However, the damaging effects extend far beyond the liver, impacting the brain, pancreas, gastrointestinal tract, cardiovascular system, and immune function, all underscored by underlying inflammatory and oxidative mechanisms (Varghese and Dakhode, 2022). Therefore, understanding the molecular and cellular mechanisms disrupted by chronic alcohol consumption is of paramount importance for identifying viable counteractive strategies in mitigating its deleterious systemic health consequences.

Among several metabolic networks disrupted by chronic ethanol exposure, sulfur metabolism is highly vulnerable (Yang et al., 2006). Sulfur is an essential element, incorporated into several vital biomolecules, most notably the amino acids methionine and cysteine (Brosnan and Brosnan, 2006). Beyond their role in protein synthesis, these sulfur-containing amino acids are central to cellular homeostasis through their involvement in detoxification, redox signalling, nucleic acid and polyamine synthesis, and epigenetic regulation (Li and Zhang, 2024). The transsulfuration pathway plays a pivotal role in this system by converting methionine and homocysteine into cysteine, thereby regulating cysteine availability for glutathione (GSH) biosynthesis (Aoyama and Nakaki, 2015). As a major intracellular antioxidant, GSH maintains redox homeostasis through direct ROS scavenging and by serving as a cofactor for antioxidant enzymes (Vašková et al., 2023). Therefore, the functional efficiency of the transsulfuration pathway are critical for preserving cellular redox homeostasis and mitigating oxidant-induced injury (Sbodio et al., 2018), both of which are significantly compromised by chronic ethanol exposure.

At the intersection of methionine metabolism lies S-adenosyl-L-methionine (SAMe), a metabolite of extraordinary biological significance. Synthesized from methionine and adenosine tri-phosphate (ATP), SAMe serves as the principal methyl group donor in countless transmethylation reactions and serves as a key precursor feeding into the transsulfuration pathway (Gao et al., 2018). Following methyl donation, SAMe is converted to S-adenosylhomocysteine (SAH), which is subsequently hydrolyzed to homocysteine. Homocysteine may then be remethylated to methionine or diverted into the transsulfuration pathway to generate cysteine and, ultimately, glutathione (GSH) (Kumar et al., 2017). Through these interconnected processes, SAMe acts as a pivotal regulator of both epigenetic regulation and cellular antioxidant capacity. Notably, SAMe depletion is a characteristic feature of several liver pathologies, including alcohol-associated liver disease (ALD), leading to impaired methylation reactions and compromised GSH synthesis (Jung et al., 2013). Alcohol and its metabolites further exacerbate this disruption by inhibiting key enzymes involved in SAMe metabolism and transsulfuration pathway (Villanueva and Halsted, 2004; Kharbanda, 2013), including methionine adenosyl transferase (MAT), cystathionine β-synthase (CBS), and cystathionine γ-lyase (CSE) (Johnson et al., 2021). These impairments collectively reduce antioxidant capacity, thereby promoting oxidative stress and inflammatory signaling. The resulting redox imbalance contributes to the activation of hepatic immune cells, such as Kupffer cells, and enhances the production of pro-inflammatory cytokines including tumor necrosis factor-α (TNF-α) and interleukin-6 (IL-6), accelerating tissue injury and disease progression (Heberlein et al., 2014; Yan et al., 2023; Grigore et al., 2025; Nordback et al., 2025). Given this mechanistic framework, therapeutic strategies aimed at restoring SAMe availability and transsulfuration pathway function represent a promising approach to mitigating alcohol-induced organ damage. Preclinical studies have demonstrated that SAMe supplementation can reduce liver injury markers, improve antioxidant status, and attenuate inflammation and fibrosis progression (Baden et al., 2024). Similarly, our recent study demonstrated that SAMe reverses ethanol-induced developmental toxicity in zebrafish embryos by modulating oxidative stress and glutathione homeostasis (Nandagopal et al., 2026). Although glutathione precursors such as N-acetylcysteine (NAC) have been widely studied in alcohol-induced tissue injury (Vendemiale et al., 2001; Ozaras et al., 2003), SAMe was selected for the present study due to its dual capacity to support methylation reactions and enhance glutathione synthesis via the transsulfuration pathway. The functionality of these pathways are also dependent on specific B-vitamins that acts as cofactors. Vitamin B6 (pyridoxal phosphate) is essential for the enzymes CBS and CSE in the transsulfuration pathway. Vitamin B12 (cobalamin) is crucial for the methionine synthase enzyme, which remethylates homocysteine (Werge et al., 2021). Vitamin B1 supports mitochondrial energy metabolism and contributes to glutathione redox cycling (Depeint et al., 2006). Deficiencies in these vitamins are most common in individuals with chronic alcohol consumption due to poor diet and impaired absorption, which can further cripple sulfur metabolism, leading to homocysteine accumulation (Hyperhomocysteinemia) and reduced GSH biosynthesis (Cravo and Camilo, 2000). Therefore, supplementation of these B-vitamins could ideally support the efficiency of remethylation and transsulfuration pathways, helping to maintain SAMe homeostasis and boost GSH production, thereby offering a complementary strategy to combat alcohol-induced damage.

While the hepatoprotective effects of SAMe in alcohol-associated liver disease (ALD) are established (Paredes et al., 1987; Mato et al., 1999; McClain et al., 2002), the novelty of the present study extends beyond monotherapy approaches by rigorously evaluating the combinational efficacy of SAMe and selected B-vitamin cofactors, with a focus on their potential to support glutathione homeostasis and redox balance during chronic ethanol exposure. This research addresses the existing gap and clinical challenge by evaluating a combinational supplementation strategy aimed at concurrently mitigating ethanol-induced injury in primary ethanol-target organs, including the liver and pancreas, thereby providing crucial translational data for developing more effective combinational therapies. In conclusion, the depletion of the major antioxidant glutathione via impairment of the transsulfuration pathway, coupled with the dysregulation of the critical methyl donor SAMe, leaves cells vulnerable to the oxidative and inflammatory cascade triggered by alcohol metabolism. Thus, the current objective was designed to evaluate the use of SAMe and relevant B-vitamins supplementation as a multi-targeted nutritional strategy to enhance antioxidant defences, supress inflammation and ultimately confer organ protection against the chronic effects of alcohol exposure.

## 2 Materials and methods

### 2.1 Chemicals and kits

Molecular biology grade ethanol was procured from Hi-Media laboratories, India. The Lieber-DeCarli ’82 Shake and Pour control and ethanol liquid diets were purchased from Bio-Serv (USA) (product numbers F1259SP and F1258SP, respectively). Kits for quantifying triglycerides (TGL), amylase (AMY), alanine aminotransferase (ALT) and aspartate aminotransferase (AST) activities were purchased from Sigma Aldrich (Bangalore, India). Enzyme linked Immuno-sorbent assay (ELISA) kits for tumour necrosis factor-alpha (TNF-α) quantification and NF-kb binding activity was purchased from Cayman chemicals, USA. Trizol, cDNA synthesis kit and SYBR green master mix for qRT-PCR was procured from Takara, Japan. S-adenosyl-L-methionine (SAMe), Vitamin B1, B6, B12, zinc acetate, trichloroacetic acid (TCA), thiobarbiturate (TBA), ferric chloride, tetramethylbenzidine (TMB), hexadecyl-trimethylammonium bromide (CTAB), sodium phosphate, hydrogen peroxide (H_2​_O_2_​), 5,5-dithiobis-(2-nitrobenzoic acid (DTNB), potassium phosphate and NNDP sulphate were sourced from Sigma-Aldrich (Bangalore, India).

### 2.2 Animal husbandry

All the animal studies were conducted in accordance with accepted guidelines for animal research (ARRIVE) and were authorised by the Vellore Institute of Technology’s Institutional Animal Ethical Committee (IAEC) (Approval no. VIT/IAEC/25/Nov27/15). Animal subjects were sourced from the Biogen Laboratory (Bengaluru, India) and were maintained in the animal housing facility in a controlled temperature range of 21–24^0^C with a 12:12 h light-dark cycle. Before starting the trial, the animals were quarantined for a week and were fed with standard chow feed and water. 8–10 weeks old C57BL/6J mice (male) weighing above 20 g were selected and used for the present study.

### 2.3 Leiber-DeCarli liquid feed model (*ad libitum*)

**FIGURE 1:**
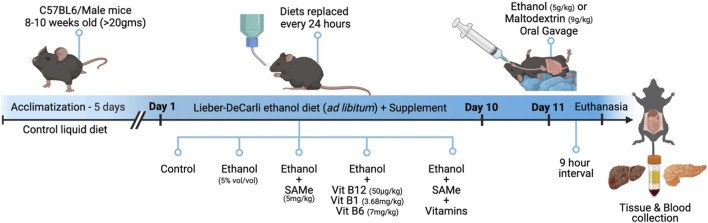
Schematic overview of the experimental protocol, including animal grouping, ethanol exposure and supplementation regimen followed in the study.

NIH-NIAAA (National Institute on Alcohol Abuse and Alcoholism) standardised chronic-plus-single-binge ethanol feeding model was adapted, to model the complex nature of acute-on-chronic alcohol-associated liver injury observed clinically (Bertola et al., 2013). The experimental protocol begins with a 5-day acclimatisation of all the animals to the control liquid diet (*ad libitum*) and tube feeding. A total of thirty animals (N = 30) were utilized in this study and were randomly allocated into five distinct experimental groups (n = 6 per group) and were treated for ten consecutive days as detailed: (i) Control fed with calorie balanced control liquid diet, (ii) ethanol (5% v/v) fed with ethanol liquid diet (Bertola et al., 2013), (iii) ethanol + SAMe (5 mg/kg) (Song et al., 2003), (iv) ethanol + Vitamins B6 (7 mg/kg), B12 (50 μg/kg), B1 (3.68 mg/kg) (Reeves et al., 1993; NRC, 1995; Reeves, 1997) and (v) ethanol + SAMe + Vitamins. SAMe, B-vitamins and ethanol were mixed thoroughly into their respective liquid diets immediately prior to administration to the animals. The control and ethanol-containing liquid diets were formulated to be isocaloric, ensuring complete caloric equivalence between the dietary groups. Animals consistently consumed the complete daily portion of both the control and ethanol-containing liquid diets, enabling accurate estimation of ethanol intake based on the known ethanol concentration in the diet, has thereby eliminated the need for metabolic cage monitoring. Subsequently, on the morning of the eleventh day, all the ethanol-treated groups were gavaged with a single intragastric dose of ethanol (5 g/kg) and the control groups were gavaged with isocaloric maltose dextrin solution (9 g/kg). Nine hours later, animals were euthanized using carbon dioxide (CO_2_) in a controlled chamber with a volume displacement rate of 20% per minute. Serum was separated from the collected blood samples. Liver and pancreatic tissues samples were carefully excised, snap frozen and stored at −80^0^ C for further experimentations (Figure 1).

### 2.4 Body weight assessment

The body weight of all animals was monitored using a digital scale (Kent Scientific, USA) on alternating days throughout the experimental period. To normalize the data and account for individual variations, the initial weight of each animal group was considered as 100%. All subsequent weight measurements were reported as percentage change from this baseline. This method allows for a consistent assessment of the effects of supplementation on body weight over time.

### 2.5 ALT and AST enzyme activity measurement

To evaluate the degree of hepatic injury, the serum enzyme activities of key hepatic enzymes, including alanine aminotransferase (ALT) and aspartate aminotransferase (AST) were assessed. The enzyme activities were measured spectrophotometrically using commercially sourced ALT and AST activity assay kit (Sigma-Aldrich, India), according to the manufacturer’s protocol. Enzyme activities were expressed as units per litre (U/L).

### 2.6 Amylase activity assay

Amylase (AMY) activity assay kit by Sigma-Aldrich (Bangalore, India) was employed to assess the serum AMY activity in the extracted serum sample. Essentially, kit contains substrate ethylidene-pNP-G7 linked with a chromophore, which will be broken down upon interaction with the α-amylase, producing coloured p-nitrophenol. A spectrophotometer (BioTek, USA) is used to measure this increase in colour intensity as the reaction proceeds at a specific wavelength of 405 nm. AMY activity levels are displayed in units of U/L.

### 2.7 Quantification of triglycerides

TGL level in the serum was quantified using a TGL assay kit from Sigma-Aldrich (Bangalore, India). The kit employs the Glycerol Phosphate Oxidase-Peroxidase (GPO-POD) method to enzymatically hydrolyse triglycerides, culminating in the production of a quantifiable quinoneimine dye. The intensity was absorbed at 546 nm using an microplate reader (BioTek, USA) and the TGL levels were represented as mg/DL.

### 2.8 Lipid peroxidation assessment

Malondialdehyde (MDA), a stable end product of lipid peroxidation during oxidative stress and cellular damage was measured using the Thiobarbituric Acid Reactive Substances (TBARS) assay (Aguilar Diaz De Leon and Borges, 2020). Shortly, 100 µL of homogenate and TCA (5% w/v) were combined, and rested on ice for 10 min 100 μL of TBA (0.67% w/v) was added to the mixture and centrifuged for 10 min at 4 °C at 5000 rpm. Aliquoted supernatant was boiled in a 95 °C water bath and cooled to room temperature for recording the absorbance at 535 nm. Obtained results were normalised with protein and expressed as µM of MDA per mg of protein.

### 2.9 Measurement of nitric oxide production

Griess reagent (Sigma Aldrich, USA) was utilized to determine the concentration of NO (Bryan and Grisham, 2007). Briefly, a 1:1 mixture of tissue homogenate and griess reagent were incubated in the dark for 15 min at RT. The intensity of the colour developed was recorded at 540 nm using microplate reader (BioTek, USA). Absorbance values were plotted against the standard curve to calculate the levels of NO. The final values were normalized to the protein content of the tissue and the results were expressed as µM of NO per mg of protein.

### 2.10 Measurement of hydrogen sulphide

H_2_S released from the hepatic and pancreatic tissues of mice were evaluated by methylene-blue assay (Smith and Pluth, 2023). Tissue homogenate prepared from potassium phosphate buffer (ice-cold) is used for this reaction. Initially, zinc acetate (1% w/v, 150 µL) is used to precipitate the gaseous hydrogen sulphide to zinc sulphide, preventing it from escaping (Amiti et al., 2019). 20 nM of NNDP sulphate prepared in 7.2 M HCl and 30 mM of FeCl3 prepared in 1.2 M of HCl were added and incubated in dark for 20 min to facilitate the reaction. Finally, TCA (10% w/v) was added to the suspension and centrifuged at 4,000 rpm for 10 min. Optical density values were measured at 670 nm, compared with the standard (NaHS), normalised with the protein content and finally expressed as µM of H_2_S per mg of protein.

### 2.11 Reduced glutathione quantification

GSH quantification was performed using established Ellman Assay, where GSH reacts specifically with the Ellman’s reagent (DTNB) to yield 2-nitro-5-mercapto-benzoic acid (TNB), which produces a concentration-dependent yellow chromophore quantifiable by spectrophotometry (Vuolo et al., 2022). In short, 450 µL of 50 mM phosphate buffer (pH 7.4) and 1 mL of 60 µM DTNB were mixed with 150 µL of homogenate. After transferring the reaction mixture to a microplate reader (BioTek, USA), the absorbance at 412 nm was determined. Values were normalised and reported as µM of reduced glutathione per mg of protein (Latha Laxmi and Tamizhselvi, 2025).

### 2.12 Measurement of myeloperoxidase activity

Neutrophils are primary responders to inflammation, which can be studied by quantifying the activity of myeloperoxidase, an enzyme abundantly present in their granules (Khan et al., 2018). Centrifuged pellet from the homogenized tissues of liver and pancreas were resuspended in 50 mM phosphate buffer containing CTAB (0.5% w/v) (Brahadeeswaran et al., 2024). Sample is subjected to rigorous freeze-thaw cycles, followed by a 40 s sonication procedure to ensure an complete enzyme release. This suspension is then centrifuged (10,000 g) for 5min at 4 °C. Finally, MPO assay is initiated by mixing equal volumes of the resulting supernatant with the TMB substrate and incubated for 110 s. Reaction was then stopped by adding sulphuric acid (0.18M) and the absorbance was read at 450 nm with a microplate reader. Enzyme activity was expressed as fold change in comparison with the control.

### 2.13 Histopathological examination

Liver and pancreatic tissues were duly fixed in formalin (10%) solution to preserve structural integrity and prevent decomposition. These tissues were subjected to multiple ethanol washes of ascending concentrations (70%, 90%, 100%) to dehydrate them. Xylene is used to replace the ethanol as a paraffin miscible solvent. Subsequently, tissues were infiltrated and embedded by molten paraffin wax and microtome was employed to prepare tissue sections of 5 µm thickness. Mounted tissue sections were stained with Haematoxylin and Eosin (H&E). The structural integrity and cellular morphology of the liver and pancreatic tissues were examined using light microscopy. Histopathological evaluation of tissue sections was performed by a pathologist blinded to the experimental groups. Tissue injury was graded using a semi-quantitative four-point ordinal severity scale, wherein scores were assigned as follows: Normal Histology = 0, Mild alteration = 1, Moderate alteration = 2, Severe alteration = 3. For liver sections, scoring criteria included alterations in hepatocyte morphology, sinusoidal architecture, portal triad integrity, central vein appearance, and inflammatory cell infiltration. Pancreatic sections were scored based on the integrity of islets of Langerhans, organization of pancreatic acini, evidence of periductal inflammation, pancreatic duct alterations, and interstitial inflammatory infiltration.

### 2.14 Reverse transcription - quantitative polymerase chain reaction

Following homogenization, total RNA was isolated from liver and pancreatic tissues using Trizol reagent. The yield and integrity of the isolated RNA was evaluated with a Nanodrop (ThermoFisher Scientific). One microgram of the purified RNA was then reverse transcribed to complementary DNA (cDNA) using PrimeScript RT Reagent Kit (Perfect Real Time) (TaKara, Japan). Gene expression was subsequently quantified by performing quantitative real-time PCR (qRT-PCR) by using the cDNA as template. The amplification was carried out using SYBR Premix Ex Taq™ II (Tli RNaseH Plus) master mix (TaKara, Japan) and primers as mentioned in Table. 1. Relative gene expression was quantified using the comparative 2^−ΔΔCT^ method, with glyceraldehyde 3-phosphate dehydrogenase (*Gapdh*) serving as the housekeeping gene for normalization.

**TABLE 1:** Primer sequences used for PCR.

Gene name	Forward primer	Reverse primer	Accession number
*Gclc*	ACA​CCT​GGA​TGA​TGC​CAA​CGA​G	CCT​CCA​TTG​GTC​GGA​ACT​CTA​C	NM_010295
*Gpx1*	CGC​TCT​TTA​CCT​TCC​TGC​GGA​A	AGT​TCC​AGG​CAA​TGT​CGT​TGC​G	NM_008160
*Nos2*	GAG​ACA​GGG​AAG​TCT​GAA​GCA​C	CCA​GCA​GTA​GTT​GCT​CCT​CTT​C	NM_010927
*Cyp2e1*	AGG​CTG​TCA​AGG​AGG​TGC​TAC​T	AAA​ACC​TCC​GCA​CGT​CCT​TCC​A	NM_021282
*Gapdh*	CAT​CAC​TGC​CAC​CCA​GAA​GAC​TG	ATG​CCA​GTG​AGC​TTC​CCG​TTC​AG	NM_008084

### 2.15 TNF-α quantification by ELISA

To quantify the levels of the pro-inflammatory cytokine TNF-α, Enzyme-linked Immunosorbent Assay (ELISA) was performed using the tissue homogenates of liver and pancreas. Tissue samples were homogenized in sodium phosphate buffer and subsequently centrifuged at 4 °C. Resulting supernatant was utilized to perform the ELISA by following the manufacturer’s (Cayman chemical, USA) protocol. The expression levels of the cytokine were depicted in pg/mL.

### 2.16 Isolation of nuclear proteins

Quantifying the DNA-binding activity of nuclear NF-κB p65 provides a functional measure of NF-κB activation and offers insight into the transcriptional regulation of inflammatory genes encoding cytokines and chemokines (Lawrence, 2009). Nuclear extraction is crucial to understand the NF-κB DNA-binding activity. This procedure involves separation of nucleus from the cell, while maintaining the integrity of the nuclear proteins. A commercially available nuclear extraction kit (Cayman chemical, USA) was used for this process. To prevent any further protein modifications, the homogenized tissues were resuspended in ice cold PBS containing phosphatase inhibitors. Homogenate is centrifuged at a low speed (300g, 5min) to pellet down the intact nuclei. Nuclear pellet were resuspended in hypotonic solution and detergent to facilitate osmotic shock and the suspension was centrifuged at high speed (1,4000 g, 30 s). Nuclear proteins are extracted from the pellet containing pure nuclear fraction, with the help of nuclear extraction buffer, which also has proteasome inhibitors to prevent nuclear protein degradation (Sundar and Tamizhselvi, 2020).

### 2.17 NF-κB DNA-binding activity assay

Prepared nuclear extracts were used to assess NF-κB p65 DNA-binding activity as a measure of NF-κB activation in response to cellular stimuli, using a simple and efficient ELISA-based NF-κB (p65) Transcription Factor Assay Kit from Cayman Chemical (USA). 10 μg of nuclear proteins were added to each well, which are pre-coated with a specific double-stranded oligonucleotide comprising the NF-κB binding sequence (5′-GGGACTTTCC-3′). This sequence, captures any activated NF-κB (p65) present in a nuclear protein extract. A primary antibody that can particularly bind to the p65 subunit is added to the wells. Adding, HRP-conjugated secondary antibody will help in binding of the antibodies. Finally, HRP catalysing substrate is added to produce a coloured product, which is measured at 450 nm. Results are presented as optical density (OD) values recorded at 450 nm.

### 2.18 Statistical analysis

The experimental outcomes are projected as mean ± SD. To estimate any statistically significant differences between the groups, one-way ANOVA with Bonferroni’s multiple comparisons were employed. All the quantitative analysis was performed with the help of GraphPad prism 9.0 software. Statistical significance was asserted only if the *p* values were less than 0.05. In the results, significance is indicated by symbols corresponding to the calculated p-values: # or * for *p* < 0.05, ## or ** for *p* < 0.01, and ### or *** for *p* < 0.001.

## 3 Results

### 3.1 SAMe and B-vitamins supplementation improve body weight index during ethanol exposure

**FIGURE 2:**
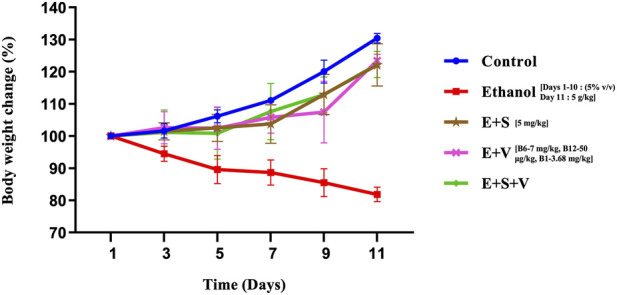
Effects of ethanol and supplementation on body weight index. Body weight measurements were recorded across all experimental groups on days 1, 3, 5, 7, 9, 11 and were expressed as percentage change relative to baseline. Data are presented as mean ± SD (n = 6). Experimental groups included Control (C), ethanol (E), ethanol + SAMe (E + S), ethanol + Vitamins (E + V), and ethanol + SAMe + Vitamins (E + S + V).

The ethanol-treated group exhibited a progressive, time-dependent reduction in mean body weight, with an approximate 18% decrease observed by day 11 relative to day 1 (Figure 2). In contrast, control animals demonstrated a gradual increase in mean body weight, resulting in an approximate 30% increase by day 11 compared to day 1. The supplementation groups did not show any significant changes in their mean body weight during the initial phase of the study (up to day 5). Thereafter, body weight in the supplemented groups increased steadily, resulting in an approximate 22%–23% gain by day 11 relative to day 1.

### 3.2 SAMe and B-vitamins supplementation attenuate ethanol-induced alterations in ALT, AST, amylase, and triglycerides

**FIGURE 3:**
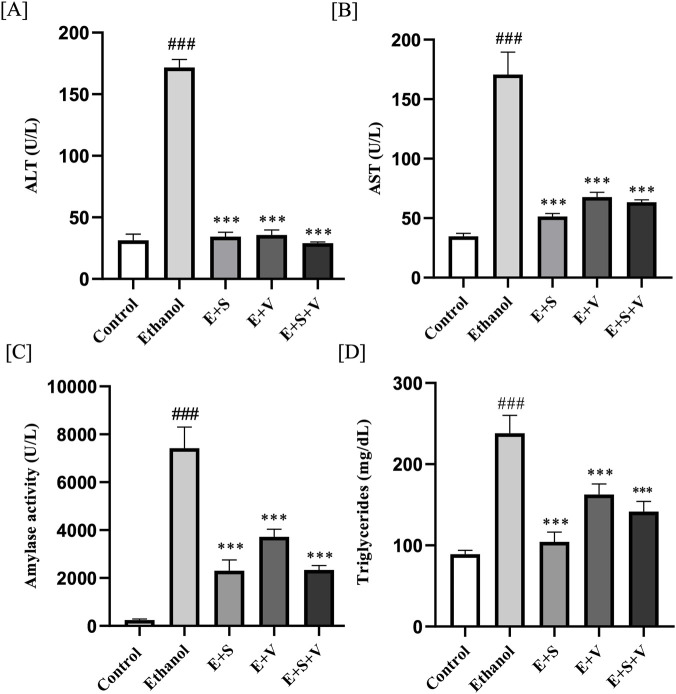
Effects of ethanol and supplementation on serum enzyme activities of ALT **(A)** AST **(B)** and Amylase **(C)** along with Triglycerides levels **(D)** measured across treatment groups. Data are presented as mean ± SD (n = 6). Statistical significance is indicated as * or #*p* < 0.05, ** or ##*p* < 0.01, *** or ###*p* < 0.001 (# vs. control and * vs. ethanol). Experimental groups included Control (C), ethanol (E), ethanol + SAMe (E + S), ethanol + Vitamins (E + V), and ethanol + SAMe + Vitamins (E + S + V).

Figures 3A–C illustrates that serum enzymatic activities of ALT, AST and AMY were substantially elevated (multi-fold) in the ethanol treated group compared to the control (*p* < 0.001). All the supplementation groups exhibited statistically significant reversal of these serum enzyme activities (*p* < 0.001) compared to ethanol. Serum TGL levels were significantly elevated in the ethanol group compared with controls (*p* < 0.001) (Figure 3D). In contrast, supplementation with SAMe and B-vitamins resulted in a significant reduction in TGL levels relative to the ethanol group (*p* < 0.001).

### 3.3 SAMe and B-vitamins supplementation reduce ethanol-induced lipid peroxidation

**FIGURE 4:**
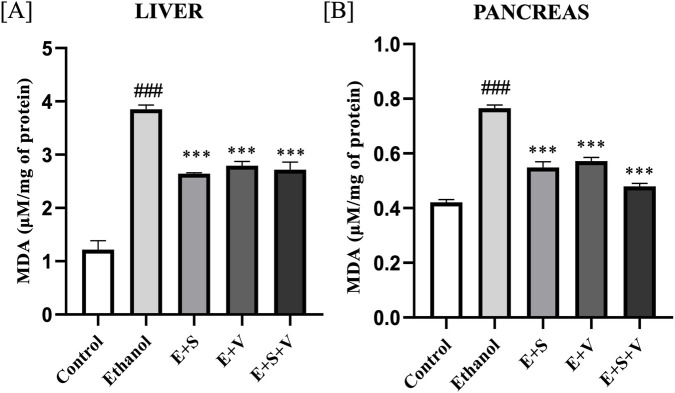
Impact of ethanol and supplementation on MDA levels (lipid peroxidation) measured in liver **(A)** and pancreas **(B)** across treatment groups. Data are presented as mean ± SD (n = 6). Statistical significance is indicated as * or #*p* < 0.05, ** or ##*p* < 0.01, *** or ###*p* < 0.001 (# vs. control and * vs. ethanol). Experimental groups included Control (C), ethanol (E), ethanol + SAMe (E + S), ethanol + Vitamins (E + V), and ethanol + SAMe + Vitamins (E + S + V).

Malondialdehyde (MDA), a stable end product of lipid peroxidation (Kasdallah-Grissa et al., 2006) was significantly elevated in both liver (Figure 4A) and pancreatic tissues (Figure 4B) of the ethanol treated group compared to the control (*p* < 0.001). The administration of SAMe and B-vitamins significantly alleviated MDA levels (*p* < 0.001) in both the liver and pancreatic tissues.

### 3.4 SAMe and B-vitamins supplementation mitigate ethanol-induced upregulation of nitric oxide and *Nos2*

**FIGURE 5:**
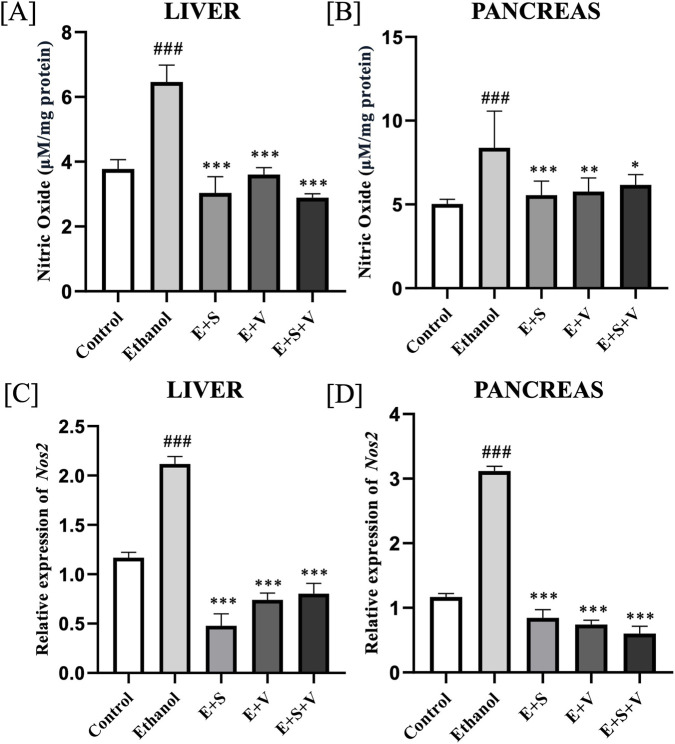
Effects of ethanol and supplementation on NO production measured in liver **(A)** and pancreas **(B)** across treatment groups. Modulation of *Nos2* gene expression by ethanol and supplementation in liver **(C)** and pancreas **(D)** measured across treatment groups. Data are presented as mean ± SD (n = 6). Statistical significance is indicated as * or #*p* < 0.05, ** or ##*p* < 0.01, *** or ###*p* < 0.001 (# vs. control and * vs. ethanol). Experimental groups included Control (C), ethanol (E), ethanol + SAMe (E + S), ethanol + Vitamins (E + V), and ethanol + SAMe + Vitamins (E + S + V).

Analysing the expression of *Nos2* and nitric oxide (NO) levels, revealed a marked elevation of these nitrosative stress markers in both liver (Figures 5A,C) and pancreatic tissues (Figures 5B,D) of the ethanol treated group (*p* < 0.001) compared to the control. Both the monotherapies and co-supplementation of SAMe and B-vitamins effectively downregulated these markers (*p* < 0.001).

### 3.5 SAMe and B-vitamins supplementation counteracted ethanol-induced suppression of H_2_S synthesis

**FIGURE 6:**
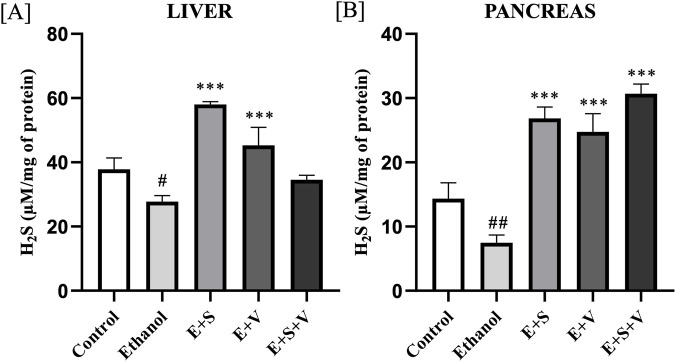
Impact of ethanol and supplementation on H_2_S levels measured in liver **(A)** and pancreas **(B)** across treatment groups. Data are presented as mean ± SD (n = 6). Statistical significance is indicated as * or #*p* < 0.05, ** or ##*p* < 0.01, *** or ###*p* < 0.001 (# vs. control and * vs. ethanol). Experimental groups included Control (C), ethanol (E), ethanol + SAMe (E + S), ethanol + Vitamins (E + V), and ethanol + SAMe + Vitamins (E + S + V).

Ethanol group showed significant depletion of H_2_S levels in the liver (*p* < 0.05) and pancreas (*p* < 0.01) compared to control (Figures 6A,B). In the pancreatic tissues, all supplementation groups significantly enhanced the H_2_S levels (*p* < 0.001) compared to ethanol group. Interestingly, only the monotherapies with SAMe and B-vitamins significantly restored the liver H_2_S levels (*p* < 0.001), whereas all the supplementation groups effectively restored the pancreatic H_2_S levels (*p* < 0.001) compared to ethanol group.

### 3.6 SAMe and B-vitamins supplementation reverse ethanol-induced impairment of GSH biosynthesis and expression of *Gclc* and *Gpx1*

**FIGURE 7:**
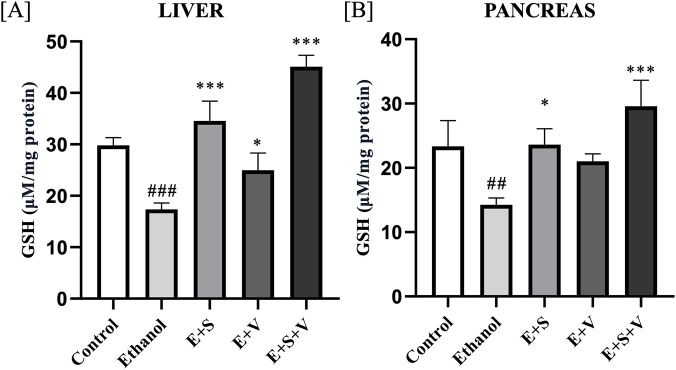
Effects of ethanol and supplementation on GSH concentration measured in liver **(A)** and pancreas **(B)** across treatment groups. Data are presented as mean ± SD (n = 6). Statistical significance is indicated as * or #*p* < 0.05, ** or ##*p* < 0.01, *** or ###*p* < 0.001 (# vs. control and * vs. ethanol). Experimental groups included Control (C), ethanol (E), ethanol + SAMe (E + S), ethanol + Vitamins (E + V), and ethanol + SAMe + Vitamins (E + S + V).

Antioxidant potential of the supplementation was assessed by quantifying the molecular concentrations of glutathione (GSH), which revealed a significant depletion of GSH levels (*p* < 0.001) in both the liver and pancreatic tissues of the ethanol treated group (Figures 7A,B) compared to the control. Supplementation groups containing SAMe exhibited significant restoration of GSH levels (*p* < 0.001) in both the organs, while B-vitamins groups only improved the liver GSH levels (*p* < 0.01) compared to the ethanol group.

**FIGURE 8:**
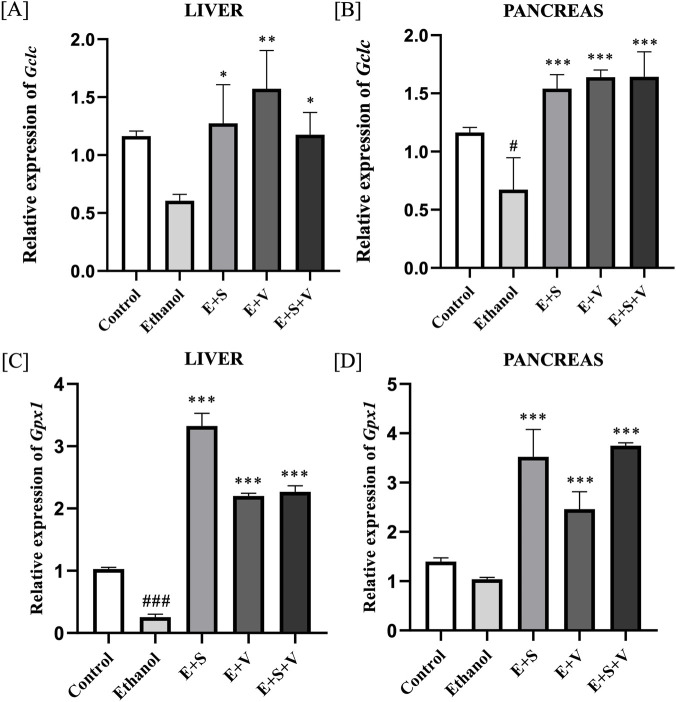
Effects of ethanol and supplementation on *Gclc* and *Gpx1* gene expression in liver **(A,C)** and pancreas **(B,D)** Measured across treatment groups. Data are presented as mean ± SD (n = 6). Statistical significance is indicated as * or #*p* < 0.05, ** or ##*p* < 0.01, *** or ###*p* < 0.001 (# vs. control and * vs. ethanol). Experimental groups included Control (C), ethanol (E), ethanol + SAMe (E + S), ethanol + Vitamins (E + V), and ethanol + SAMe + Vitamins (E + S + V).

Figures 8A,B show that ethanol exposure significantly downregulated *Gclc* expression in both the liver and pancreas (*p* < 0.01). In the liver, supplementation resulted in a moderate upregulation of *Gclc* expression (*p* < 0.05), relative to the ethanol group. In the pancreas, all supplementation groups demonstrated highly significant upregulation (*p* < 0.01) of *Gclc* expression compared with the ethanol treated group. Ethanol exposure resulted in a significant downregulation of *Gpx1* expression in liver tissue (*p* < 0.001) compared to control. In contrast, pancreatic *Gpx1* expression showed a modest, but not statistically significant, reduction compared with control. Notably, all the supplementation groups exhibited statistically significant (*p* < 0.001) upregulation of *Gpx1* expression in both liver and pancreatic tissues as observed in Figures 8C,D.

### 3.7 SAMe and B-vitamins attenuate ethanol-induced histopathological damage in liver and pancreas tissues

**FIGURE 9:**
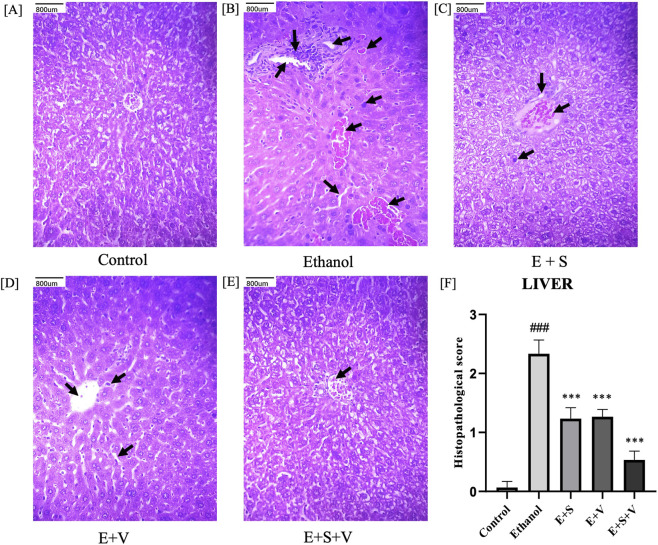
Impact of ethanol and supplementation on hepatic tissue architecture and corresponding histological scores **(F)** assessed across treatment groups. Histopathological images **(A–E)** Illustrate H&E-stained tissue section of a single representative animal from each treatment group (Scale bar = 800 µm). Arrows indicate pathological changes, including interstitial edema, neutrophil infiltration, sinusoidal dilation, hepatocyte vacuolation, erythrocyte congestion, inflammation and necrosis. Data are presented as mean ± SD (n = 6). Statistical significance is indicated as * or #*p* < 0.05, ** or ##*p* < 0.01, *** or ###*p* < 0.001 (# vs. control and * vs. ethanol). Experimental groups included Control (C), ethanol (E), ethanol + SAMe (E + S), ethanol + Vitamins (E + V), and ethanol + SAMe + Vitamins (E + S + V).

Control mice exhibited well-preserved hepatic architecture, whereas the ethanol treated group showed pronounced hepatic injury characterized by interstitial edema, neutrophil infiltration, sinusoidal dilation, hepatocyte vacuolation, erythrocyte congestion, inflammation and necrosis as shown in (Figures 9A–E). These pathological changes were reflected in significantly higher liver histological injury scores in the ethanol group compared with controls (*p* < 0.001) (Figure 9F). Supplementation with SAMe or B-vitamins alone improved the hepatic cellular architecture. Only, SAMe and B-vitamins co-supplementation resulted in most pronounced attenuation of ethanol-induced pathological features, as evidenced by improved hepatocyte morphology, preserved sinusoidal structure, and a near-normal appearance of the portal triad. Consistent with these observations, liver histopathological injury scores were significantly reduced (*p* < 0.001) in the co-supplementation group compared with the ethanol treated group.

**FIGURE 10:**
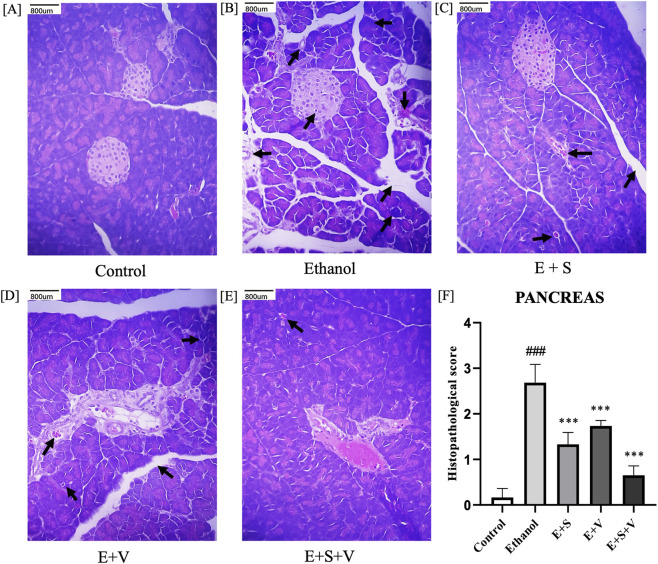
Impact of ethanol and supplementation on pancreatic tissue architecture and corresponding histological scores **(F)** assessed across treatment groups. Histopathological images **(A–E)** Illustrate H&E-stained tissue section of a single representative animal from each treatment group (Scale bar = 800 µm). Arrows indicate pathological changes, including disrupted islets of Langerhans, peri-ductal inflammation, interstitial edema, neutrophil infiltration, degenerated acinar cells and necrosis. Data are presented as mean ± SD (n = 6). Statistical significance is indicated as * or #*p* < 0.05, ** or ##*p* < 0.01, *** or ###*p* < 0.001 (# vs. control and * vs. ethanol). Experimental groups included Control (C), ethanol (E), ethanol + SAMe (E + S), ethanol + Vitamins (E + V), and ethanol + SAMe + Vitamins (E + S + V).

Histological examination of pancreatic tissues revealed marked ethanol-induced pathological alterations, including disruption of the islets of Langerhans, degenerative changes in pancreatic acini, peri-ductal inflammation, neutrophil infiltration, interstitial edema and necrosis (Figures 10A–E). Among the treatment groups, SAMe and B-vitamins co-supplementation, notably attenuated these histopathological features, as evidenced by preservation of pancreatic cellular architecture and morphology that closely approximated control tissues. Consistent with these qualitative observations, pancreas histopathological injury scores were significantly reduced (*p* < 0.001) in the co-supplementation group compared with the ethanol treated group (Figure 10F).

### 3.8 SAMe supplementation mitigate ethanol-induced neutrophil infiltration by suppressing MPO activity

**FIGURE 11:**
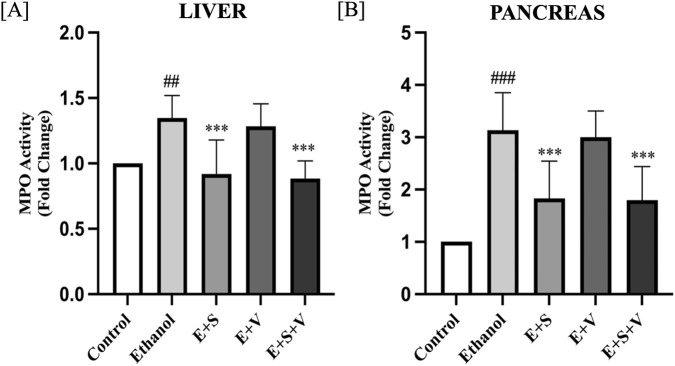
Effects of ethanol and supplementation on MPO activity (Neutrophil Infiltration) measured in liver **(A)** and pancreas **(B)** across treatment groups. Data are presented as mean ± SD (n = 6). Statistical significance is indicated as * or #*p* < 0.05, ** or ##*p* < 0.01, *** or ###*p* < 0.001 (# vs. control and * vs. ethanol). Experimental groups included Control (C), ethanol (E), ethanol + SAMe (E + S), ethanol + Vitamins (E + V), and ethanol + SAMe + Vitamins (E + S + V).

Ethanol exposure resulted in a significant increase in the liver and pancreas MPO activity (*p* < 0.001) compared to the control, as shown in Figures 11A,B. SAMe supplementation effectively mitigated this ethanol-induced increase in MPO activity (*p* < 0.001) in both liver and pancreatic tissues. In contrast, B-vitamin supplementation alone did not significantly affect the liver and pancreas MPO levels. In the co-supplementation group, the reduction in MPO activity in both the liver and pancreas, was comparable to the effect observed with SAMe alone (*p* < 0.001), indicating that the protective effect against neutrophil infiltration was primarily driven by SAMe.

### 3.9 SAMe and B-vitamins supplementation reduce ethanol-induced upregulation of *Cyp2e1* gene expression

**FIGURE 12:**
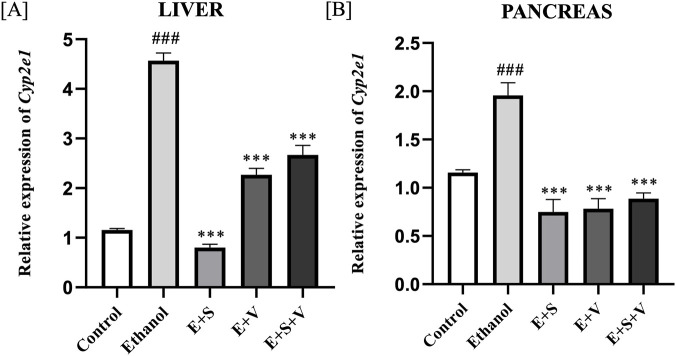
Effects of ethanol and supplementation on *Cyp2e1* gene expression in liver **(A)** and pancreas **(B)** measured across treatment groups. Data are presented as mean ± SD (n = 6). Statistical significance is indicated as * or #*p* < 0.05, ** or ##*p* < 0.01, *** or ###*p* < 0.001 (# vs. control and * vs. ethanol). Experimental groups included Control (C), ethanol (E), ethanol + SAMe (E + S), ethanol + Vitamins (E + V), and ethanol + SAMe + Vitamins (E + S + V).

Gene expression analysis revealed a marked upregulation of *Cyp2e1* in the ethanol treated group, with approximately fourfold and twofold increases observed in the liver and pancreas, respectively, compared with control group (*p* < 0.001). All supplementation groups exhibited significant downregulation (*p* < 0.001) of *Cyp2e1* expression in both organs (Figures 12A,B).

### 3.10 SAMe and B-vitamins supplementation mitigate ethanol-induced production of TNF-α and activation of NF-κB

**FIGURE 13:**
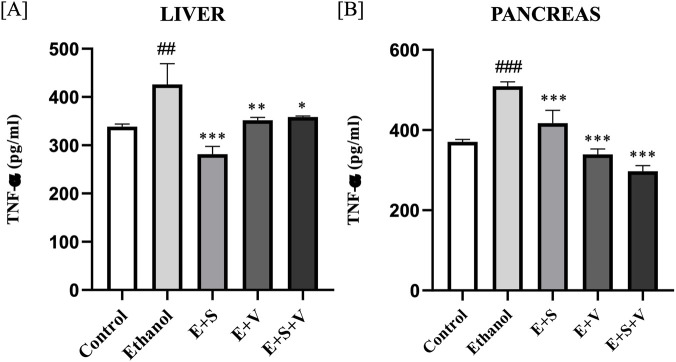
Impact of ethanol and supplementation on TNF-α production measured in liver **(A)** and pancreas **(B)** across treatment groups. Data are presented as mean ± SD (n = 6). Statistical significance is indicated as * or #*p* < 0.05, ** or ##*p* < 0.01, *** or ###*p* < 0.001 (# vs. control and * vs. ethanol). Experimental groups included Control (C), ethanol (E), ethanol + SAMe (E + S), ethanol + Vitamins (E + V), and ethanol + SAMe + Vitamins (E + S + V).

Quantitative analysis of TNF-α demonstrated a significant elevation in the ethanol treated group in both the liver (*p* < 0.01) and pancreas (*p* < 0.001). In the liver, SAMe supplementation produced pronounced reduction in TNF-α levels (*p* < 0.001), while B-vitamins (*p* < 0.01) and co-supplementation groups (*p* < 0.05) resulted in moderate decreases in liver TNF-α levels (Figure 13A) compared to the ethanol group. All the supplemented groups displayed significantly reduced the pancreas TNF-α levels (*p* < 0.001) (Figure 13B) compared to the ethanol group.

**FIGURE 14:**
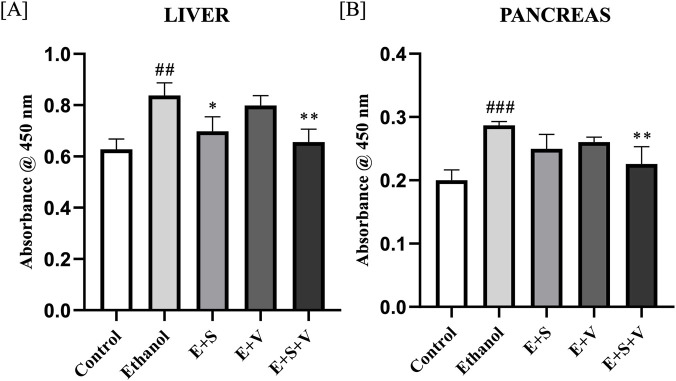
Impact of ethanol and supplementation on NF-κB DNA-binding activity measured in liver **(A)** and pancreas **(B)** across treatment groups. Data are presented as mean ± SD (n = 6). Statistical significance is indicated as * or #*p* < 0.05, ** or ##*p* < 0.01, *** or ###*p* < 0.001 (# vs. control and * vs. ethanol). Experimental groups included Control (C), ethanol (E), ethanol + SAMe (E + S), ethanol + Vitamins (E + V), and ethanol + SAMe + Vitamins (E + S + V).

Figures 14A,B demonstrate the significant increase in NF-κB (p65) DNA-binding activity in the ethanol treated group within the liver (*p* < 0.01) and pancreas (*p* < 0.001) compared to the control. While monotherapies exhibited mild (*p* < 0.05) to no significant reduction, in liver and pancreas NF-κB (p65) DNA-binding activity, SAMe and B-vitamins co-supplementation group significantly attenuated (*p* < 0.01) the NF-κB (p65) DNA-binding activity in both the liver and pancreas, compared to the ethanol group.

## 4 Discussion

The present study evaluated the hepato-pancreatic protective effects of combined S-adenosyl-L-methionine (SAMe) and B-vitamins supplementation in a murine model of chronic ethanol exposure. To closely recapitulate the clinical features of acute-on-chronic alcohol-associated liver injury observed clinically, a NIAAA - standardized feeding paradigm was employed. This model reliably reproduces key metabolic, inflammatory, and oxidative disturbances observed in human disease. Sustained availability of SAMe has been shown to influence sulfur amino acid metabolism by supporting transsulfuration flux (Stipanuk, 2004), in part through maintaining homocysteine availability and modulating remethylation dynamics, including effects linked to MTHFR regulation (McCaddon and Miller, 2023). In parallel, B-vitamins serve as indispensable cofactors for critical enzymes within the transsulfuration pathway, notably CBS and CSE, which govern cysteine production and downstream glutathione biosynthesis (Werge et al., 2021). Collectively, this mechanistic interplay provides a strong rationale for the combined supplementation strategy investigated in this study. The observed improvements in antioxidant markers and redox-related outcomes suggest that coordinated support of methylation and transsulfuration pathways may contribute to enhanced cellular resilience against ethanol-induced oxidative and inflammatory stress. These findings highlight the therapeutic potential of SAMe and B-vitamins co-supplementation as a targeted metabolic strategy to mitigate ethanol-associated hepato-pancreatic injury. While both SAMe and B-vitamins are involved in one-carbon metabolism and methylation pathways, contributing to antioxidant defenses, glutathione biosynthesis, and metabolic homeostasis, their exact organ-specific mechanisms of action in the liver and pancreas remain to be elucidated in future studies. Importantly, the novelty of the present study lies in the combinational use of SAMe and B-vitamins to potentially support methionine metabolism, enhance glutathione biosynthesis, and strengthen antioxidant defenses, thereby, providing integrated protection against alcohol-induced liver and pancreatic injury.

Ethanol is a source of empty calories that provides energy without essential nutrients, chronic uptake of which is often evidenced by a decrease in body weight (Lieber, 1991). Hepatic metabolism prioritizes ethanol over other macronutrients, such as fats and carbohydrates, which leads to a decrease in their absorption and storage (Goldman et al., 2025). This metabolic shift, together with the ethanol-induced impairment of hepatic metabolic capacity, may have contributed to the observed weight loss (Figure 2). The improvement in body weight observed in the supplementation groups may be an indicative of enhanced metabolic efficiency, nutrient utilization and energy homeostasis even under ethanol-induced metabolic stress. Hepatic injury associated with chronic ethanol exposure was evaluated by quantifying the activities of key clinical enzymes in serum, including ALT, AST, and AMY (Lee et al., 2023; User, 2017). Elevated activities of ALT and AST, key aminotransferases involved in amino acid metabolism, indicate compromised hepatocellular membrane integrity, potentially resulting from ethanol-induced toxicity (Moriles et al., 2024). SAMe and B-vitamins supplementation proved their hepato-protective effect by significantly lowering the serum levels of ALT and AST. Donohue, 2007 has reported that ethanol induced hepatic-steatosis severely damages the ideal metabolic events at liver, leading to excessive build-up of fat. Factors like increased fatty acid synthesis, decreased fatty acid oxidation and impaired lipid export resulting from hepatic alcohol metabolism significantly influence the serum TGL levels. Elevated serum TGL levels is a key indicator of steatotic liver and a common consequence of long-term heavy drinking (Khemichian and Donovan, 2023). Hypertriglyceridemia, disrupts the cardiovascular system by promoting atherosclerosis and also it is one of the major contributors of acute pancreatitis (Feingold, 2022). Pancreatic acini leaks abnormal levels of the digestive enzyme, AMY into the bloodstream when they are injured or inflamed under ethanol exposure (Wang et al., 2020). The observed alleviation of both TGL and AMY levels in the SAMe and B-vitamins supplementation groups (Figures 3C,D) demonstrates the hypo-triglyceridemic and pancreo-protective effects of these supplements respectively. This significant mitigation of serum enzyme activities, including ALT, AST, and AMY, together with the reduction in triglyceride levels, provide compelling evidence that the present supplementation strategy effectively mitigates ethanol-induced hepatic and pancreatic injury.

Lipid peroxidation, an autocatalytic chain reaction initiated by ROS from ethanol metabolism, produces mutagenic and genotoxic byproducts like MDA. These ROS oxidize polyunsaturated fatty acids (PUFAs) in cell membranes, thereby producing free radicals that propagate the cycle, resulting in the destruction of cell membranes (Ayala et al., 2014). SAMe and vitamin supplementation significantly mitigated this lipid peroxidation, as evidenced by reduced MDA levels (Figures 4A,B), likely reflecting the restoration of key cellular antioxidant defences as discussed previously. Inflammatory stimulus elicited by the body in response to ethanol, led to the strong upregulation of the gene that encodes for iNOS enzyme. Upon activation, *Nos2* produces aberrant levels of NO, a crucial molecule in immune defence. (Figures 5A–D). Although NO is essential for routine vital processes like vasodilation and neurotransmission, an overabundance of NO can have severe pathological consequences. Elevated levels can react with superoxide to produce peroxynitrite, which can subsequently lead to direct DNA damage and mutations (Iwakiri and Kim, 2015). Hence, the downregulation of these biomarkers was of critical clinical importance for managing inflammation and oxidative stress, a therapeutic objective that was effectively achieved through this supplementation.

The observed alterations in key antioxidant molecules, including H_2_S and GSH in the liver and pancreatic tissues, following chronic ethanol exposure may reflect a disruption of the transsulfuration pathway, a metabolic route responsible for generating these molecules from sulfur-containing amino acids such as methionine and cysteine (Kabil et al., 2011; Sbodio et al., 2018). H_2_S and GSH are pivotal components of the cellular antioxidant system, essential for protecting the organs from oxidative stress and inflammation, thereby addressing the principal etiological factors underlying hepatitis and pancreatitis. The therapeutic potential of SAMe and B-vitamins supplementation is demonstrated in its apparent capacity to restore H_2_S and GSH levels, thereby supporting the maintenance of cellular redox homeostasis. Evaluating the gene expression levels of Glutamate-cysteine ligase catalytic subunit (*Gclc*), that codes for the catalytic subunit of the enzyme glutamate-cysteine ligase (GCL) is essential in understanding the genetic regulation of GSH biosynthesis (Dickinson et al., 2004; Franklin et al., 2008). Nuclear translocation is central for the transcription of genes like *Gclc* and *Gpx1*, that codes for the crucial enzymes of the antioxidant system. The observed upregulation of *Gclc* in the liver and pancreatic tissues, following supplementation suggests a potential enhancement of the cellular capacity for glutathione biosynthesis (Koide et al., 2003). Functionality and efficiency of the GSH system, was further validated by assessing the gene expression levels of glutathione peroxidase 1 (*Gpx1*) (Lubos et al., 2011). Supplementation mediated improvement of *Gpx1* gene expression may reflect a possible increase of the cellular antioxidant defence capacity, as *Gpx1* encodes a key glutathione peroxidase enzyme, which is involved in the detoxification of ROS, including hydrogen peroxide and lipid hydroperoxides, thereby potentially contributing to attenuation of oxidative stress (Quintana-Cabrera et al., 2012; Handy and Loscalzo, 2022). However, the underlying mechanism of action for this effect is a subject of ongoing research. We acknowledge that the inclusion of an exogenous GSH treatment group would further strengthen the mechanistic interpretation of antioxidant involvement in this model and will be considered in future investigations.

Ethanol-induced organ damage is characterized by significant inflammation and neutrophil infiltration. Neutrophils, being the primary responders of inflammation, infiltrate the liver and pancreatic cells. Subsequent degranulation of these immune cells, during an inflammatory response releases an enzyme called MPO. Elevated MPO activity in the liver and pancreatic tissues of ethanol treated groups (Figures 11A,B) served as a direct indicator of inflammation and associated oxidative stress (Wang, 2018). Chronic ethanol exposure resulted in pronounced structural alterations in both liver and pancreatic tissues, including hepatocyte degeneration, necrosis, and disruption of normal pancreatic architecture, reflects the extensive cellular and inflammatory stress induced by ethanol (Figures 9, 10). Supplementation-mediated restoration of cellular architecture and histological integrity, along with reduced neutrophil infiltration (as indicated by MPO activity) in the both the liver and pancreas highlights the tissue-protective capacity of SAMe and B-vitamins co-supplementation. This observed efficacy of the supplement is attributable to its ability to provide antioxidant support, modulate anti-inflammatory signalling, and facilitate cellular repair and regeneration.

A significant upregulation of *Cyp2e1*, a well-established biomarker of chronic ethanol exposure (Wang et al., 2023), was observed in both liver and pancreatic tissues (Figures 12A,B). The inherent inefficiency of the corresponding enzyme, cytochrome P450 2E1 in metabolising ethanol, results in significant production of ROS and harmful byproducts that are directly linked to inflammation, lipid peroxidation, and subsequent organ damage. The substantial downregulation of *Cyp2e1* observed in the supplementation groups may reflect a corresponding reduction in CYP2E1 enzyme expression. This effect could be associated with the antioxidative properties of the supplementation, which may attenuate the ROS-driven positive feedback loop that otherwise sustains *Cyp2e1* induction (Jin et al., 2013). As a well-known methyl donor, SAMe may also transcriptionally modulate *Cyp2e1* gene expression through methylation at the gene’s promoter region, representing an potential alternative mechanism of regulation (Ye et al., 2021).

Compromised intestinal permeability following ethanol exposure facilitates the leakage of gut bound bacterial endotoxin, lipopolysaccharide (LPS) into the circulation (Gao et al., 2011). This prompts the activation of *Kupffer* cells resulting in massive secretion of the TNF-α, a pro-inflammatory cytokine that initiates a vicious cycle of inflammation by coordinating the immune responses (Slevin et al., 2020). SAMe supplementation was associated with a significant reduction in the liver and pancreas TNF-α levels, which may reflect a transcriptional modulation potentially linked to its methyl-donor capacity (Figures 13A,B). Research indicates that SAMe also suppresses the transcription of the *TNF-*α gene, by preventing the nuclear translocation of NF-κB (Moon et al., 2010). Likewise, B-vitamins supplementation may contribute to the attenuation of TNF-α expression, potentially through their roles as essential cofactors in metabolic pathways that influence inflammatory and redox homeostasis (Ehmedah et al., 2019; Rakić et al., 2023). Overproduction of ROS during ethanol metabolism serves as a direct trigger for the activation of the NF-κB pathway. Ethanol-induced redox imbalance may further amplify this process by promoting the activation and nuclear translocation of redox-sensitive transcription factors, thereby facilitating the transcriptional induction of pro-inflammatory genes. This process may contribute to the maintenance of a persistently heightened pro-inflammatory state, thereby exacerbating tissue injury and promoting progressive organ damage. SAMe and B-vitamins co-supplementation reduced the NF-κB (p65) DNA-binding activity in the liver and pancreas (Figures 14A,B), which may be attributable to improved antioxidant status and enhanced ROS neutralization, thereby potentially attenuating a key upstream driver of NF-κB activation (Morgan and Liu, 2010). The reduction in NF-κB (p65) DNA-binding activity in SAMe and B-vitamins co-supplementation group indicates an overall attenuation of inflammatory signalling in response to chronic ethanol exposure (Tak and Firestein, 2001; Yu et al., 2020). While these findings support the protective role of SAMe and B-vitamin supplementation, the present study has certain limitations that should be considered. Specifically, organ-specific mechanisms such as pancreatic calcium signaling were not evaluated, and tissue levels of triglycerides (TGL), fatty acid ethyl esters (FAEEs), and SAMe were not directly measured in the liver and pancreas. Addressing these factors in future investigations will provide deeper mechanistic insight and allow a more precise evaluation of the protective effects of SAMe and B-vitamin supplementation against alcohol-induced hepato-pancreatic injury.

## 5 Conclusion

**FIGURE 15:**
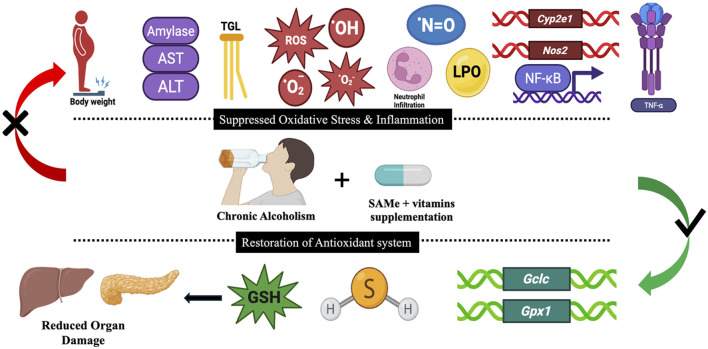
Graphical overview illustrating the central findings and their physiological implications during ethanol-induced hepato-pancreatic injury, followed by supplementation-mediated organ protection.

Collectively, the findings of the present study provide compelling evidence that SAMe and corresponding, co-factorial B-vitamins effectively re-establish the synthesis and functionality of endogenous antioxidants like GSH and H_2_S. This combinational intervention significantly attenuated multiple biochemical and molecular markers associated with ethanol-induced organ injury, including pro-inflammatory signalling (NF-κB and TNF-α), gaseous mediators (*Nos2* and NO), enzymatic markers (ALT, AST, AMY), alcohol-metabolizing cytochromes (*Cyp2e1*), lipid peroxidation and tissue damage (MPO). By re-establishing redox homeostasis and conferring a significant anti-inflammatory effect, SAMe and B-vitamins co-supplementation has notably improved the overall health of liver and pancreas (Figure 15). Therefore, the present study has established a promising *de novo* combinational supplementation strategy for counteracting the physiological damage and long-term health sequelae associated with chronic alcohol exposure. Nonetheless, a deeper mechanistic elucidation is warranted to elucidate the mechanism of action of this supplementation, thereby solidifying the basis for future interventions.

## Data Availability

The original contributions presented in the study are included in the article/supplementary material, further inquiries can be directed to the corresponding author.

## Glossary

ALD: Alcohol-associated liver disease
ALT: Alanine aminotransferase
AMY: Amylase
AST: Aspartate aminotransferase
ATP: Adenosine Tri-Phosphate
CBS: Cystathionine β-synthase
cDNA: Complementary Deoxyribonucleic acid
CSE: Cystathionine γ-lyase
CTAB: Cetyltrimethylammonium bromide
*Cyp2e1*: Cytochrome P450 2E1
*Gapdh*: Glyceraldehyde 3-phosphate dehydrogenase
GCL: Glutamate-cysteine ligase
*Gclc*: Glutamate–cysteine ligase catalytic subunit
GPO-POD: Glycerol Phosphate Oxidase-Peroxidase
*Gpx1*: Glutathione peroxidase 1
GSH: Glutathione
**H**_**2**_ **S**: Hydrogen Sulphide
IL-6: Interleukin-6
iNOS: Inducible nitric oxide synthase
Keap1: Kelch-like ECH-associated protein 1
LPO: Lipid peroxidation
LPS: Lipopolysaccharide
MAT: Methionine Adenosyl transferase
MDA: Malondialdehyde
MPO: Myeloperoxidase
NaHS: Sodium hydrosulphide
NF-κB: Nuclear factor kappa B
NNDP: N, N-dimethyl-p-phenylenediamine sulphate
NO: Nitric Oxide
*Nos2*: Nitric Oxide Synthase 2
PUFAs: Polyunsaturated fatty acids
qRT-PCR: Quantitative real-time PCR
ROS: Reactive oxygen species
SAH: S-adenosyl Homocysteine
SAMe: S-adenosyl-L-methionine
TBA: Thiobarbiturate
TCA: Trichloroacetic acid
TGL: Triglycerides
TNF-α: Tumour necrosis factor alpha
